# Physico-Chemical and Antifungal Properties of a Trypsin Inhibitor from the Roots of *Pseudostellaria heterophylla*

**DOI:** 10.3390/molecules23092388

**Published:** 2018-09-18

**Authors:** Xixi Cai, Xiaoli Xie, Nanyan Fu, Shaoyun Wang

**Affiliations:** 1 The Key Lab of Analysis and Detection Technology for Food Safety of the MOE, College of Chemistry, Fuzhou University, Fuzhou 350108, China; caixx_0123@163.com (X.C.); nanyan_fu@fzu.edu.cn (N.F.); 2 Institute of Food and Marine Bio-Resources, College of Biological Science and Technology, Fuzhou University, Fuzhou 350108, China; xiaoli18850225337@163.com

**Keywords:** trypsin inhibitor, *Pseudostellaria heterophylla*, fluorescence spectroscopy, circular dichroism, antifungal

## Abstract

Plant peptidase inhibitors play essential roles in the defense systems of plants. A trypsin inhibitor (PHTI) with a molecular mass of 20.5 kDa was isolated from the fresh roots of the medicinal herb, *Pseudostellaria heterophylla*. The purification process involved ammonium sulfate precipitation, gel filtration chromatography on Sephadex G50, and ion-exchange chromatography on DEAE 650M. The PHTI contained 3.7% α-helix, 42.1% β-sheets, 21.2% β-turns, and 33% disordered structures, which showed similarity with several Kunitz-type trypsin inhibitors. Inhibition kinetic studies indicated that PHTI was a competitive inhibitor, with a K*i* value of 3.01 × 10^−9^ M, indicating a high affinity to trypsin. The PHTI exhibited considerable stability over a broad range of pH (2–10) and temperatures (20–70 °C); however, metal ions, including Fe^3+^, Ba^2+^, Mn^2+^, and Al^3+^, could inactivate PHTI to different degrees. Results of fluorescence spectroscopy and circular dichroism showed that Fe^3+^ could bind to TI with an association constant of 2.75 × 10^5^ M^−1^ to form a 1:1 complex, inducing conformation changes and inactivation of PHTI. In addition, PHTI could inhibit the growth of the phytopathogens, *Colletotrichum gloeosporioides* and *Fusarium oxysporum*, through disruption of the cell membrane integrity. The present study extended research on *Pseudostellaria heterophylla* proteins and makes PHTI an exploitable candidate as an antifungal protein for further investigation.

## 1. Introduction

Natural peptidase inhibitors (PIs) are ubiquitous in living organisms, and have been largely described in plants [1]. Generally, PIs are particularly abundant in plant reproductive and storage organs, such as seeds and tubers, and amount to 1–15% of total soluble proteins in these tissues [2]. Previous reports have shown that PIs play essential roles in the defense systems of plants against attack by insects, fungi, and other pathogenic bacteria [2,3,4]. Besides, PIs have also been described as endogenous regulators of proteolytic processes and are known to be involved in many biological functions, such as anti-microbial infections, blood coagulation, platelet aggregation, and anti-carcinogenesis [5,6].

PIs can be divided into four classes (serine-, metallo-, cysteine-, and aspartyl-PIs) based on the family of peptidases with which they interact. Serine-PIs are the largest group among the four classes, which are active against trypsin and/or chymotrypsin-like enzymes [7]. Serine-PIs are further typed into Kunitz, Bowman-Birk, cereal, and thaumati-like inhibitors [8,9,10], among which, the Kunitz-type trypsin inhibitors (KTIs) show the highest affinity to trypsin. KTIs commonly contain four cysteine residues forming two disulfide bridges and mainly consist of β-sheets with a molecular mass of about 20 kDa [11]. Previous studies have revealed that KTIs have plasticity in their structure, which allows them to interact with peptidases with different specificities simultaneously [12].

The tuberous root of *Pseudostellaria heterophylla* (*Caryophyllaceae*) is a type of staple traditional Chinese medicine, which has been recorded in Chinese Pharmacopoeia with multiple medicinal effects, such as tonifying the spleen, nourishing vitality, moistening the lung, and improving appetite [13]. *P. heterophylla* is rich in polysaccharide [14,15], saponin [16], and cyclopeptides [17], and they exert multiple physiological functions, such as immunoregulation, antioxidant, anticancer, and so on. In contrast, although the protein content reaches up to 17% (dry weight), there are seldom reports on the proteins of the roots of *P. heterophylla*. A KTI with antifungal activity and a novel lectin isolated from *P. heterophylla* roots were reported [18]. However, the report mainly concerned the purification process and intrinsic trypsin inhibitory and hemagglutinating activity of the proteins. The description on the inhibition kinetic, structural characteristics, and antifungal mechanism were deficient. In the present report, a trypsin inhibitor (PHTI) was purified from the fresh roots of *P. heterophylla.* The physical-chemical properties of PHTI and the mechanism of the structural interaction between Fe^3+^ and PHTI were studied. Furthermore, the inhibition effects of PHTI against plant pathogens were demonstrated in this study, which make it an important candidate for further investigation on its role in plant defense.

## 2. Results and Discussion

### 2.1. Purification of PHTI

**Figure 1:**
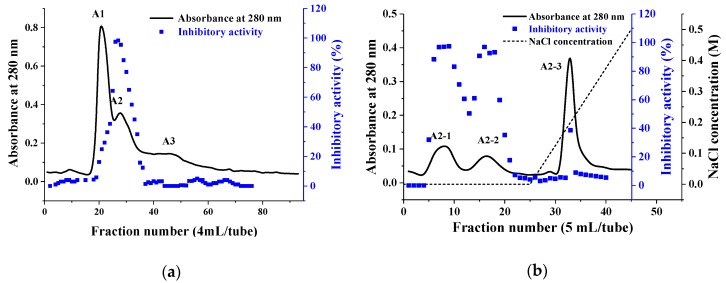
Purification of trypsin inhibitor (PHTI) from the roots of *P. heterophylla*. (**a**) Gel filtration chromatography on a Sephadex G50 column. (**b**) Ion exchange chromatography on a DEAE-650M column.

**Figure 2:**
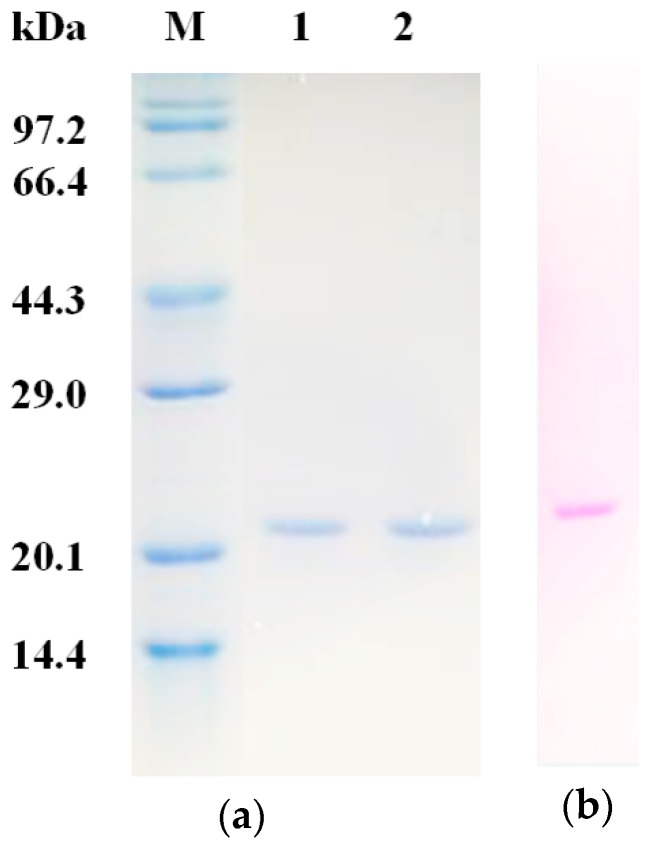
SDS-PAGE of PHTI. (**a**) Lanes: M, molecular mass marker; 1 and 2, PHTI with and without addition of beta-mercaptoethanol stained by Coomassie brilliant blue. (**b**) PHTI stained by periodic acid-schiff (PAS) reagent.

Chromatography was employed for the purification of trypsin inhibitor from the roots of *P. heterophylla*. As shown in Figure 1a, the dialyzed sample after 20–80% ammonium sulfate precipitation was divided into three fractions after Sephadex G-50 gel filtration chromatography and only fraction A2 displayed trypsin inhibitory activity (Figure 1a). Subsequently, fraction A2 was further fractionated by ion exchange chromatography on a DEAE-650M column (Figure 1b). Fraction A2-1 with 95% trypsin inhibitory activity was collected, which presented a single band on SDS-PAGE under non-reducing and reducing conditions, with an apparent molecular mass of 20.5 kDa (Figure 2a). Besides, the purified inhibitor was confirmed to be glycoprotein by SDS PAGE with periodic acid-schiff (PAS) staining (Figure 2b) and the carbohydrate content was determined to be 14.59 ± 0.47% through the anthrone-sulfuric acid method. About 65.4 mg of PHTI were obtained from 20 g of the fresh roots of *P. heterophylla*. The specific activity of the purified PHTI was determined to be 27,721.7 U/mg, with a purification fold of 15.2 and a recovery of 39.4%. The purification steps are summarized in Table 1.

### 2.2. Characterization of PHTI

#### 2.2.1. N-Terminal Amino Acid Sequences of PHTI

The N-terminal amino acids sequences of PHTI were determined to be Phe-Val-Val-Asp-Leu-Asp-Gly-Asp-Pro-Leu (FVVDLDGDPL) by Edman degradation, and were then submitted to the NCBI-BLAST database for similarity analysis. Results showed that PHTI presented a high similarity to several plant Kunitz-type trypsin inhibitors, such as inhibitors from *Leucaena leucocephala* and *Mucuna pruriens* (Table 2).

#### 2.2.2. Circular Dichroism (CD) Spectrum Analysis

**Figure 3:**
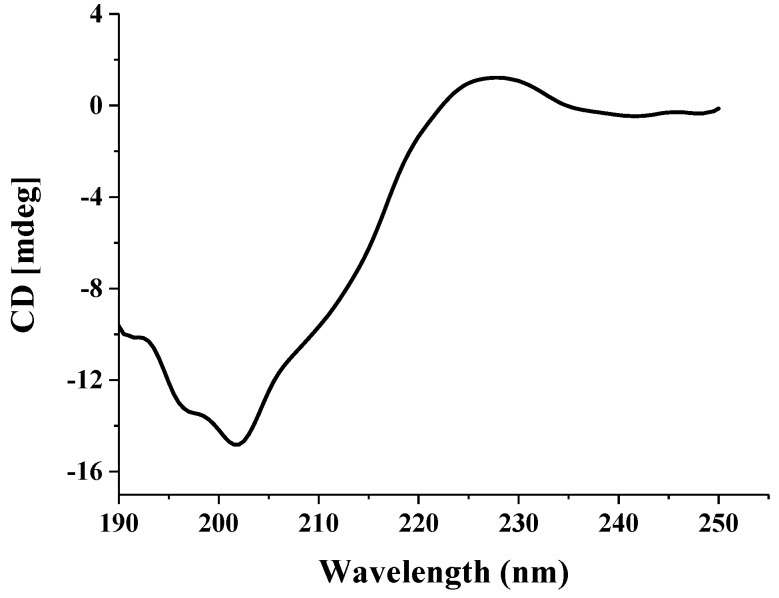
Circular dichroism spectrum of PHTI in distilled water.

The CD spectrum of PHTI was characterized by a weak positive peak near 225 nm and a negative peak around 203 nm, respectively (Figure 3). PHTI was composed of 3.7% α-helix, 42.1% β-sheets, 21.2% β-turns, and 33% disordered structures, as calculated by CDPro software. Similar inhibitors had been reported. A trypsin inhibitor from *Cassia leiandra* seeds was composed of 35% β-sheets, 14% β-turns, and 50% disordered structures [19], and the trypsin inhibitor from *Acacia plumosa* was composed of 42% β-sheets, 21% β-turns, and 37% of disordered structures [20]. Previous reports showed that KTIs typically possess few α-helix structures and 12 antiparallel β-sheets connected by long loops [21]. Disordered structures might provide flexibility to certain inhibitors to inhibit enzymes of different classes [22]. Above all, PHTI was considered to belong to Kunitz-type trypsin inhibitors and held the massive β-sheets as structural homology.

#### 2.2.3. Trypsin Inhibition Kinetics of PHTI

**Figure 4:**
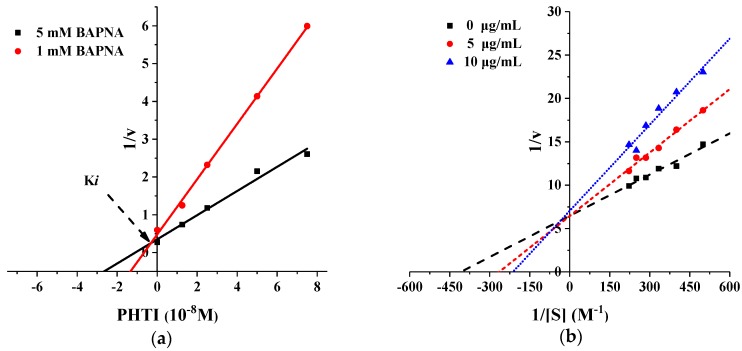
Inhibition kinetics of PHTI. (**a**) Dixon plot analysis. (**b**) Lineweaver–Burk plot analysis.

To determine the PHTI inhibition kinetic against trypsin, the Dixon and Lineweaver-Burk double reciprocal plot were employed. The initial rates of reaction of PHTI against trypsin using Nα-Benzoyl-dl-arginine 4-nitroanilide hydrochloride (BAPNA) as the substrate followed the Michaelis-Menten equation. The lines in the Dixon diagrams (Figure 4a) and Lineweaver-Burk (Figure 4b) were all intersected in the y-axis, indicating that PHTI was a competitive inhibitor, with a K*i* value of 3.01 × 10^−9^ M, which presented a high affinity between trypsin and PHTI. Kinetic studies on PHTI were in agreement with the reports for KTIs from the seeds of *Inga laurina* [23] and *Butea monosperma* [24], with a K*i* of 6 × 10^−9^ M and 1.2 × 10^−9^ M, respectively.

#### 2.2.4. Stability of Inhibitory Activity against Trypsin

##### Thermal and pH Stability

**Figure 5:**
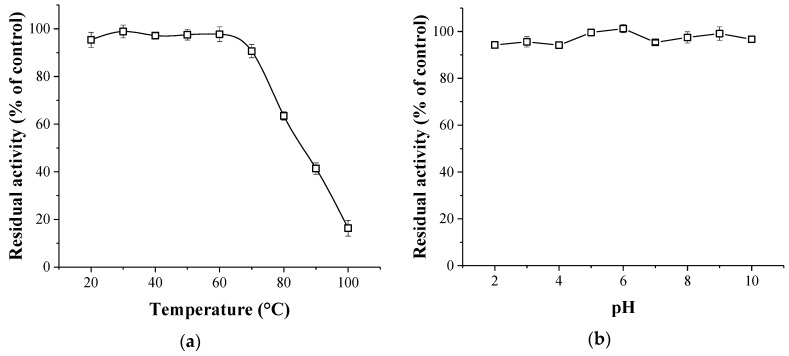
Thermal stability (**a**) and pH stability (**b**) of PHTI.

As shown in Figure 5a, trypsin inhibitory activity of PHTI was well retained after being incubated at 20–70 °C for 30 min. However, the inhibitory activity was decreased by 36.5% at 80 °C. Only 16.3% of the inhibitory activity remained after the 100 °C treatment. The thermal stability of PHTI was similar to that of KTIs from *Albizia amara* Boiv [25] and *Archidendron ellipticum* seed [2]. In addition, the inhibitory activity of PHTI was not sensitive to pH over the range of 2–10 (Figure 5b). Similar results were reported for KTIs from *Entada acaciifolia* (Benth.) seeds [26], *Catanduva* seeds [27], and *Inga laurina* (SW.) Willd seeds [23].

##### Effect of Metal Ions

Metal ions could induce changes of proteins in the conformation and thus affect the function and structure of the proteins [28]. The effects of metal ions on the trypsin inhibitory activity of PHTI were studied in the presence of different metal ions. As shown in Table 3, no notable changes in inhibitory activity were observed when concentrations of Na^+^ and K^+^ reached up to 20 mM. The addition of Cu^2+^ slightly enhanced the inhibitory activity of PHTI. Ba^2+^, Zn^2+^ Mn^2+^, and Al^3+^ had negative effects on the trypsin inhibitory activity of PHTI, with a 40–60% loss of activity at 20 mM. Among the tested ions, Fe^3+^ presented the strongest inhibition effect. With the addition of 20 mM Fe^3+^, only 9.4% trypsin inhibitory activity remained. Similarly, the trypsin inhibitor, API, from *Albizia amara* Boiv showed enhanced activity with the addition of Cu^2+^ and reduced activity in the presence of Mn^2+^, Ba^2+^, and Fe^3+^ [25]. Al^3+^ was also reported to inhibit the activity of trypsin inhibitor from *Moringa oleifera* leaves [29].

#### 2.2.5. Effect of Fe^3+^ on the Conformation Change of PHTI

**Figure 6:**
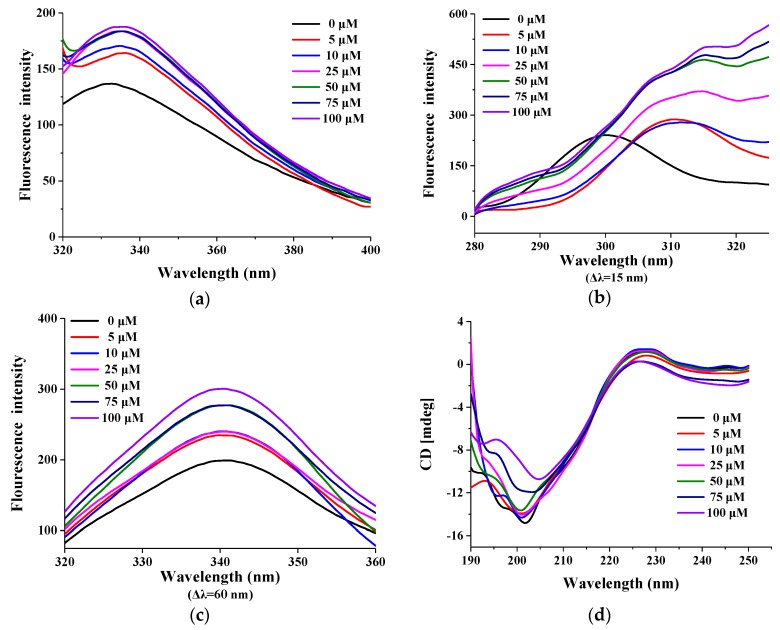
Intrinsic fluorescence spectra (**a**) and synchronous fluorescence spectra (**b**,**c**) of PHTI with Fe^3+^ (0, 5, 10, 25, 50, 75, and 100 μM, respectively). (**d**) Far-UV CD spectra of PHTI recorded in the presence of different Fe^3+^ concentrations in deionized water.

For further investigation of the mechanism of the Fe^3+^-mediated inhibition towards PHTI, both fluorescence spectroscopy and CD spectroscopy were conducted. As shown in Figure 6a, the intrinsic fluorescence intensity of PHTI increased significantly with the increase of the Fe^3+^ concentration, and a slight red shift of the maximum emission fluorescence peak (about 4 nm) appeared. The synchronous fluorescence spectra of PHTI in the presence of Fe^3+^ are also shown in Figure 6. The synchronous fluorescence was characteristic of tyrosine residues (Tyr) and tryptophan residues (Trp) at Δλ of 15 and 60 nm between the excitation and emission wavelengths, respectively [30]. The fluorescence intensities of both Tyr and Trp increased significantly with the addition of the Fe^3+^ solution (Figure 6b,c), indicating a conformation change of PHTI and the exposure of tyrosine and tryptophan residues in the presence of Fe^3+^ [31]. Furthermore, an obvious red shift (about 22 nm) of the maximum emission wavelength at the wavelength interval of 15 nm was observed, suggesting an increase in hydrophilicity around the tyrosine residues [31]. Fe^3+^ could bind to TI with an association constant of 2.75 × 10^5^ M^−1^ to form a 1:1 complex, according to the Stern-Volmer equation analysis.

Conformation changes of PHTI were analyzed by CD spectroscopy (Figure 6d). A negative peak appeared around 203 nm and a positive peak near 225 nm gradually shifted towards zero with the increase of Fe^3+^ concentrations. As the concentration of Fe^3+^ increased, the maximal ellipticity underwent a decline from 195 to 205 nm. However, the proportions of α-helix, β-sheets, β-turns, and disordered structures (calculated by CDPro) of PHTI in the presence of Fe^3+^ at various concentrations were highly consistent with PHTI (Data not shown), indicating that the addition of Fe^3+^ at the tested concentration had no effect on the secondary structure of PHTI. Therefore, we speculated that Fe^3+^ could mediate the inactivation of PHTI by changing the higher conformation rather than the secondary structure of PHTI.

### 2.3. Antifungal Activity of PHTI

**Figure 7:**
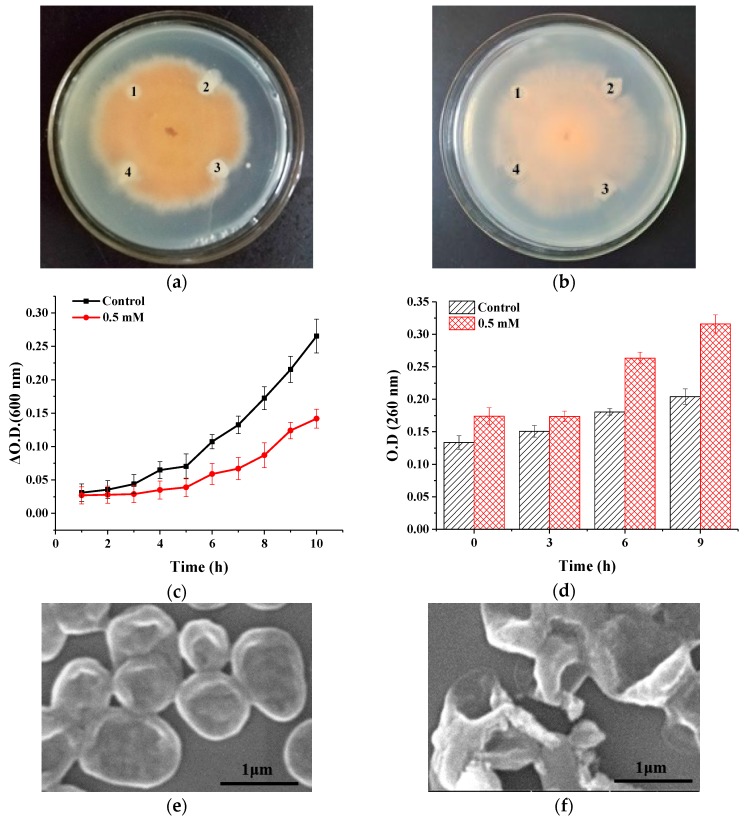

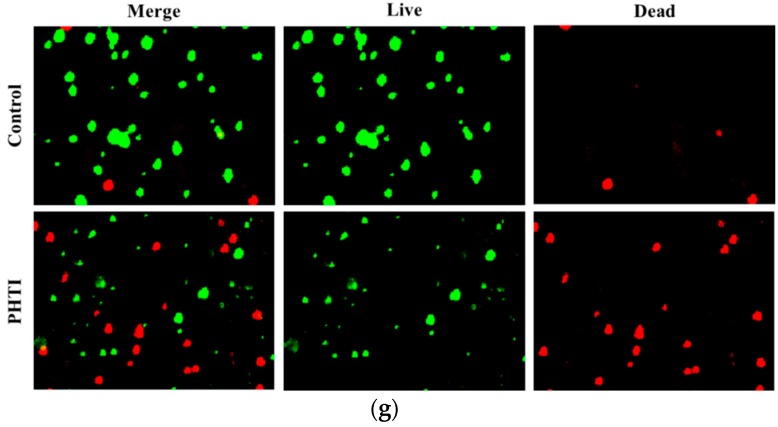
Antifungal activity of PHTI. Filter paper method for antifungals active against *C. gloeosporioides* (**a**) and *F. oxysporum* (**b**) ((1) Deionized water; (2) 0.1 mM of PHTI; (3) 0.25 mM of PHTI; (4) 0.5 mM of PHTI.) (**c**) Growth kinetics of *C. gloeosporioides*. (**d**) Total nucleotide leakage from *C. gloeosporioides* treated with 0.5 mM of PHTI. (**e**,**f**) Scanning electron microscopy (SEM) images of *C. gloeosporioides* treated with 0 and 0.5 mM of PHTI, respectively. (**g**) *C. gloeosporioides* viability visualized by fluorescence staining.

PIs, serving as a kind of defense proteins in plants, have shown inhibition of plant pathogenic fungi. Fusarium oxysporum f.sp. cubense and Colletotrichum gloeosporioides Penz are two kinds of phytopathogens of *P. heterophylla* [32]. In this work, the antifungal activity of PHTI against *C. gloeosporioides* (Figure 7a) and *F. oxysporum* (Figure 7b) were evaluated. Compared with the control disk (1), disks with 0.1 mM, 0.25 mM, and 0.5 mM of PHTI showed obvious crescents, indicating the growth inhibition of fungi. As shown in Figure 7c, 0.5 mM of PHTI could significantly lead to prolongation in the lag phase of *C. gloeosporioides* in 10 h and distinctly inhibit its growth. Trypsin inhibitors from different sources possess antifungal activity to different degrees. The antifungal activity of PHTI was comparable to the reported trypsin inhibitor from *Blighia sapida* seeds [33], while inhibitors from *Psoralea corylifolia* L. [34] and *Albizia amara* Boiv [25] exerted superior fungi inhibition effects at a magnitude of 100 μg/mL. However, trypsin inhibitors from Chinese dull black soybeans [35] and *Vigna mungo* seeds [36] did not present any fungi inhibitory effects.

Hyphal membrane permeation is a common antifungal mode of action for many defensins [3]. To investigate the effects of PHTI on the cell membrane, *C. gloeosporioides* was treated with PHTI (0.5 mM) and the nucleic acid contents in the culture medium were determined by the absorbance at 260 nm. Results showed that the addition of 0.5 mM PHTI significantly increased the extracellular nucleic acids contents, indicating damage of the cell membrane (Figure 7d). Scanning electron microscopy (SEM) experiments were conducted to directly observe the morphological changes of *C. gloeosporioides*. The control group (Figure 7e) displayed smooth surface and integral cell morphology, while the fungi treated with 0.5 mM of PHTI were found to be clustered together without clear boundaries (Figure 7f). The cell membrane integrity changes were further confirmed by fluorescence staining. The viable bacterial cells with intact membranes were visualized as green by interaction of the Syto9 fluorescent dye. On the contrary, propidium iodide entered non-viable bacterial cells with porous membranes and combined with the bacterial nucleic acid, thus turning red fluorescence. Treatment with PHTI increased the proportion of red fluorescence (Figure 7g), further suggesting that PHTI could inhibit the growth of fungi through disrupting the cell membrane integrity. *F. oxysporum* treated with PHTI exhibited similar results (Data not shown).

## 3. Materials and Methods

### 3.1. Materials

Fresh roots of *P. heterophylla* were obtained from a *P. heterophylla* planting base in Zherong, Fujian, China. The fungal strains, *F. oxysporum* and *C. gloeosporioides,* were kindly provided by the Institute of Agricultural Bio-Resources Research, Fujian Academy of Agricultural Sciences, Fuzhou, China. Toyopearl DEAE-650M and Sephadex G50 Fine were purchased from TOSOH Co., Ltd. (Tokyo, Japan) and GE Healthcare (Gothenburg, Sweden), respectively. Trypsin and BAPNA were purchased from Shanghai Yuanye Bio-Technology Co., Ltd. (Shanghai, China). All other chemicals were of analytical grade and were purchased from Sinopharm Chemical Reagent Co., Ltd. (Shanghai, China).

### 3.2. Purification of PHTI

Fresh roots of *P. heterophylla* (20 g) were homogenized (1:7.5 *w*/*v*) with Tris-HCl buffer (50 mM pH 7.4). After extraction at 4 °C for 12 h, the homogenate was filtered through four layers of gauze and centrifuged at 10,000× *g* for 15 min at 4 °C. The supernatant was subjected to ammonium sulfate precipitation. The fraction precipitated between 20% and 80% saturation was re-dissolved in a Tris-HCl buffer (50 mM, pH 7.4) and dialyzed against the same buffer at 4 °C for 24 h.

The dialyzed sample was then applied to a Sephadex G50 column (Φ 1.6 × 100 cm) pre-equilibrated with the Tris-HCl buffer (50 mM, pH 7.4). The column was eluted with the same buffer and the flow rate was 0.4 mL/min and 10 min/tube. The absorbance at 280 nm and the trypsin inhibitory activities of the fractions were determined. At the later purification stage, anion-exchange chromatography was performed on a DEAE-650M column (Φ 1.6 × 20 cm) pre-equilibrated with the Tris-HCl buffer (50 mM, pH 7.4). The column was washed with the same buffer, and the absorbed fractions were eluted with a 0–0.5 M linear gradient of NaCl. The absorbance of the fractions was monitored and the eluted protein fractions with trypsin inhibitory activity were collected.

### 3.3. Characterization of PHTI

#### 3.3.1. SDS-PAGE

SDS-PAGE was carried out in a 12.5% separating gel and 4.0% concentrating gel under reducing and non-reducing conditions according to the method of Laemmli and Favre [37]. Proteins were then stained with a 0.1% Coomassie brilliant blue R-250 solution and PAS solution, respectively [38].

#### 3.3.2. Protein N-Terminal Sequencing

The purified protein was loaded to the SDS-PAGE and then mobilized onto a membrane of polyvinylidene difluoride (PVDF) by electroblotting. The N-terminal sequence of PHTI was determined by Edman degradation and analyzed by PPSQ-30 Data Processing (Applied Biosystems, Shanghai, China).

#### 3.3.3. CD Analysis

CD spectra were used to evaluate the secondary structures and structural changes of proteins. The secondary structures of purified PHTI dissolved in deionized water with Fe^3+^ (0 to 100 μM) were measured by Bio-Logic MOS-450 (Grenoble, France) at room temperature. The spectra were recorded from 190 nm to 250 nm, with a response time of 2 s and scan speed of 100 nm/min. Quartz cuvettes of 1 cm were used. CDPro software was used to analyze the secondary structure and the data of all CD measurements were calculated in triplicate.

#### 3.3.4. Assay of Trypsin Inhibitory Activity

The trypsin inhibitory activity was calculated by the residual hydrolysis activity of trypsin towards the substrate, BAPNA, according to the method of Birk [39]. 0.4 mL of PHTI and 2 mL of BAPNA (40 mg/mL in 3 mL of dimethyl sulfoxide, diluted with 50 mM Tris-HCl buffer containing 20 mM CaCl_2_ to 100 mL) were pre-incubated at 37 °C for 10 min, then 1 mL of trypsin (0.2 mg/mL in 1 mM HCl) was added to the mixture. One unit of inhibitory activity (U) was defined as a decrease of 0.01 of absorbance at 410 nm in relation to the control sample (without inhibitor).

#### 3.3.5. Inhibition Kinetics of PHTI

The kinetic measurements of trypsin inhibition by PHTI were conducted according to Dias et al. [19], with some modifications. 0.4 mL of PHTI (0–10 nM) was incubated with various concentrations of BAPNA (0.15, 0.2, 0.25, 0.3, 0.35, 0.4, and 0.45 mg/mL) at 37 °C. The reaction was initiated by adding 1 mL of trypsin (0.2 mg/mL in 1 mM HCl), and was stopped after 10 min by the addition of 1 mL of 33% (*v*/*v*) acetic acid. All data were determined at the initial of the reaction. The absorbance was measured at 410 nm. A Lineweaver–Burk plot was obtained by the reciprocal of the rate of the enzyme reaction (1/v) versus the reciprocal of the substrate concentration (1/[S]) in the absence and presence of PHTI. The inhibition constant (K*i*) was determined according to Dixon [40]. A K*i* value was obtained by the intersection of the lines at the *x*-axis, corresponding to the different substrate concentrations. 

#### 3.3.6. Stability Studies

The thermal stability of PHTI was determined by incubating PHTI (0.25 mg/mL) at different temperatures (20–100 °C) for 30 min. Then, samples were cooled to room temperature before the remaining activities were tested as described above. The PHTI stability at different pH was evaluated by dissolving PHTI in different buffers (pH 2–10), and were incubated at room temperature for 1 h. The remaining activities were then measured. The effects of metal ions on PHTI were also evaluated. PHTI was treated with various ions (Al^3+^, Fe^3+^, Ba^2+^, Zn^2+^, Mn^2+^, Cu^2+^, K^+^, and Na^+^) for 1 h, then the remaining activities were determined as described above. The residual activity (%) was calculated by taking the activity in the control (where PHTI was without any treatments) as 100%.

#### 3.3.7. Steady-State Fluorescence

Emission spectra of PHTI were obtained using a 970 CRT spectrophotometer (Jingke, Shanghai, China) and the samples were irradiated at an excitation wavelength of 295 nm and emission wavelengths were recorded from 290–400 nm. Changes of the intrinsic fluorescence spectra of the purified PHTI (0.25 mg/mL, 1 mL) were recorded by addition of micro aliquots of FeCl_3_ (10 mmol/L) to obtain final concentrations of Fe^3+^ ranging from 0 to 100 μM. For further investigation of the effect of Fe^3+^ ions on conformation of PHTI, the synchronous fluorescence spectra were conducted at the wavelength interval (Δλ) of 15 and 60 nm, respectively. 

### 3.4. Antifungal Activity

#### 3.4.1. Filter Paper Method

The antifungal activity of PHTI was first evaluated by the filter paper method, as described by Hong et al. [41], with some modifications. The activated mycelium was picked and placed in the center of sterile potato dextrose agar plates and cultured at 28 °C for 72 h. An aliquot of PHTI was introduced to each filter paper disk (0.6 cm in diameter), placed about 0.5 cm away from the edge of the fungal colony. The plates were incubated for another 24 h until the mycelia enveloped the blank filter paper disks.

#### 3.4.2. Growth Kinetics Assay

For the experimental work, the mother cultures of fungi were prepared by inoculating the fungi from PDA to PDB medium, and then were incubated at 180 rpm, 28 °C for 72 h. The fungi were treated with 0 and 0.5 mM PHTI in a 96 well microplate and the absorbance of each well was monitored at 1 h intervals at 600 nm using a Microplate reader. Besides, the absorbance at 260 nm in the supernatant was measured for determination of the nucleic acid leakage. The assay was tested thrice on different days [42].

#### 3.4.3. SEM Analysis

The fungi were treated with 0 and 0.5 mM of PHTI for 6 h, and were fixed in 2.5% glutaraldehyde for 12 h, washed with 0.9% NaCl for 10 min, and then dehydrated in ethanol gradient (20%, 50%, 80%, 100%). Fungi samples (5 μL) were dropped on the monocrystalline silicon and dried for 1 h, then the samples were coated by gold and the surface morphologies of the fungi were visualized under SEM [43].

#### 3.4.4. Fluorescence Based Fungi Viability Assay

The fungi were treated with 0 and 0.5 mM of PHTI for 6 h, respectively. Fungal viability was evaluated using a LIVE/DEAD BacLight viability kit (Ll7012, Thermo Scientific, Waltham, MA, USA) and imaging was done by a Laser scanning confocal microscope (AI, Nikon Instruments Inc., Tokyo, Japan) [44].

### 3.5. Statistical Analysis

All data are presented as means ± standard deviations (SDs) of three independent experiments. Statistical analysis was done using the Student’s *t* test. A value of *p* < 0.05 was considered statistically significant.

## 4. Conclusions

In summary, a Kunitz-type trypsin inhibitor PHTI with a molecular mass of 20.5 kDa was purified from fresh roots of *Pseudostellaria heterophylla*. PHTI possessed superior thermal and pH stability. Fe^3+^ (20 mM) could significantly inactivate PHTI through changing its conformation. Besides, PHTI exhibited antifungal activity against phytopathogens of *P. heterophylla* by disrupting the cell membrane integrity of fungi. The present study extends the research on proteins of *P. heterophylla*, suggesting an exploitable potential for this class of antifungal protein in the food industry and agriculture.

## Figures and Tables

**Table 1:** Summary of the purification process for PHTI and its trypsin inhibitory activity.

Fractions	Total Protein(mg)	Total Trypsin Inhibitory Activity(×10^3^ U)	Specific Activity (U/mg)	Purification Fold	Extraction Yield (%)
Crude extraction ^1^	2530.0	4600.3	1818.3	—	100.0
20–80% (NH_4_)_2_·SO_4_	1259.3	3600.5	2859.1	1.6	78.3
Sephadex G-50	190.6	2407.7	12,632.2	6.9	52.3
DEAE-650M	65.4	1813.0	27,721.7	15.2	39.4

^1^ Crude extraction was gained from 20.0 g of the fresh roots of P. heterophylla.

**Table 2:** Analysis of the N-terminal sequence of Kunitz-type inhibitor from *P. heterophylla* roots.

Content	Source	Starting Amino Acid	Primary Sequences	Identity (%)
PHTI	*Pseudostellaria heterophylla*	1	F V V D L D G D P L	-
KTI	*Leucaena leucocephala*	4	- - V D L D G D P L	80
KTI	*Mucuna pruriens*	26	FVVD T D G D - -	70
KTI	*Beta vulgaris subsp. vulgaris*	25	F I L D I D G D P L	70
KTI	*Glycine soja*	27	FV L D N E G N P L	60
KTI	*Inga vera*	3	- V V D S D G E M L	60

The N-terminal sequences listed in Table 2 are from the NCBI database (https://www.ncbi.nlm.nih.gov). The amino acids with background color mean that they are the same to the sequence of PHTI.

**Table 3:** Effect of metal ions on PHTI.

Metal Ions Concentration (mM)		AlCl_3_	FeCl_3_	BaCl_2_	ZnCl_2_	MnCl_2_	CuCl_2_	NaCl	KCl
Residual activity (% of control)	0.1	94.6 ± 1.2	96.8 ± 0.5	99.8 ± 1.7	100.0 ± 1.5	100.4 ± 0.5	99.8 ± 1.2	99.1 ± 0.7	99.8 ± 0.7
	1	82.3 ± 1.5	95.0 ± 0.2	97.9 ± 1.0	98.2 ± 1.5	99.8 ± 0.2	100.9 ± 0.2	99.8 ± 0.2	100.2 ± 1.2
	5	65.8 ± 1.2	91.2 ± 0.5	95.5 ± 1.1	96.4 ± 0.8	97.3 ± 1.0	98.1 ± 0.8	99.2 ± 0.6	98.1 ± 1.3
	10	63.9 ± 3.0	29.5 ± 1.3	77.8 ± 1.2	85.8 ± 1.7	84.4 ± 1.5	117.2 ± 1.8	97.5 ± 0.5	96.8 ± 1.7
	20	60.5 ± 0.2	9.4 ± 1.3	40.5 ± 1.4	60.2 ± 0.4	67.6 ± 0.6	106.2 ± 1.6	98.8 ± 1.0	94.4 ± 1.7

Values represent the mean ± SD of three replicates.
